# A high-fat diet: an unexpected role in preventing the metastatic seeding of colorectal cancer

**DOI:** 10.1038/s41392-020-00386-2

**Published:** 2020-11-03

**Authors:** Min Long, Wenjing Wang, Qiu Sun

**Affiliations:** State Key Laboratory of Biotherapy, Cancer Center, West China Hospital, Sichuan University and Collaborative Innovation Center for Biotherapy, 610041 Chengdu, China

**Keywords:** Cancer, Gastrointestinal cancer

Recently, Wei X et al. published a study in *Signal Transduction and Targeted Therapy*, in which they demonstrated that the peritoneal seeding of colorectal cancer (CRC) can be restrained by short-term treatment with a high-fat diet (HFD) in the early phase.^1^

Peritoneal carcinomatosis (PC), occurring nearly in the final stage of CRC, is one manifestation of metastatic CRC associated with high mortality. Tumor cells of CRC invade vessels from the primary site and travel to other locations by vessels or other means to continue growing, forming the same type of tumor as the one at the primary site.^2^ Although some progress has been made in the treatment of PC, for instance, hyperthermic intraperitoneal chemotherapy,^3^ which increases the sensitivity of cancer cells to chemotherapeutic drugs by a combination of chemotherapy and hyperthermia, the mortality of the metastatic seeding of CRC remains high. Therefore, the exploration of a more effective preventive strategy for CRC–PC is urgently needed.

**Fig. 1:**
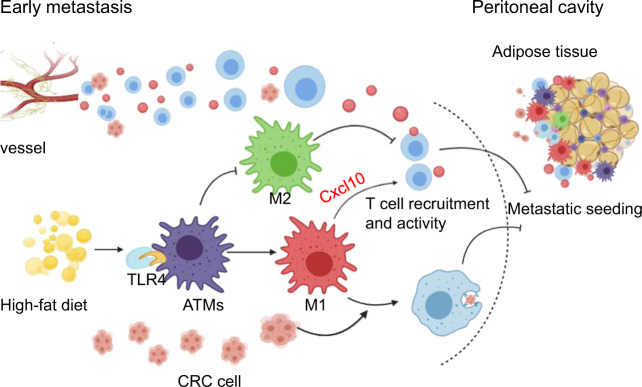
A high-fat diet (HFD) stimulates the activation of adipose tissue macrophages (ATMs) from M2 to M1 and induces proinflammatory cytokine expression in a TLR4-dependent manner to suppress colorectal cancer (CRC) cell metastasis in the early stage (Created with BioRender.com)

Clinically, patients with CRC need routine fasting and parenteral nutrition through intravenous infusion after surgery. A fat emulsion that can improve postoperative prognosis is often used to provide energy for patients after surgery, and has become an important component of parenteral nutrition. Wei X et al. established PC models in mice by intraperitoneally engrafting CRC cells to simulate the intraperitoneal dissemination of cancer cells after colorectal tumor operation, and unexpectedly found that the activation of adipose tissue macrophages (ATMs) and the tumor phagocytosis of ATMs in a TLR4-dependent manner are stimulated through a HFD (Fig. 1). The HFD inhibited the transfer of tumor cells, which are likely to be present in the visceral fat of colorectal tumors. Specifically, the consumption state of a HFD stimulates the phagocytosis of macrophages and the production of CXCL10 in a TLR4-dependent manner. CXCL10 can be activated by the M1 phenotype and promotes the recruitment of T cells to visceral fat to create a proinflammatory environment in the tumor site. Furthermore, a HFD induces ATMs from the M2-like phenotype (CD11c^−^CD206^+^) to the M1-like phenotype (CD11c^+^CD206^−^).^4^ However, in nude mice fed a HFD, adipocytes provide energy for the quick growth of metastatic cancer cells, which indicates that the suppression of the peritoneal seeding of CRC cells by HFD consumption relies on lymphocytes; that is, in mice with immune deficiency, high fat promotes PC progression, and in mice with normal immunity, high fat inhibits PC progression and prolongs survival.

Notably, the time window of HFD intervention is important. The PC model simulates the dissemination of cancer cells in the abdominal cavity of patients with early CRC after surgery. A timely and short-term HFD intervention after surgery can effectively inhibit PC progression and prolong survival in PC mice. Studies postulate that in clinical practice, high-fat interventions should be applied immediately after CRC surgery, and that adequate amounts of high fat should be included in parenteral nutrition. Further studies have demonstrated that traditional drugs, such as oxaliplatin or 5-fluorouracil, can be used in combination with a HFD to achieve a better survival rate for the prevention of CRC–PC than treatment with either drug alone. In addition, side effects can be limited when combined with a HFD, as the dose of these drugs decreases, suggesting that short-term HFD intervention with conventional drugs could be given to patients to reduce the side effects during the treatment of CRC–PC.

Overall, short-term, high-fat intervention is a simple, economical, and effective diet therapy with no side effects and has synergistic effects with conventional chemotherapy drugs. HFD treatment could be widely used in the management of CRC–PC to reduce economic pressure and the side effects during treatment. This finding overturns the traditional view that a HFD may lead to the deterioration of cancer,^5^ and provides a novel concept and potential therapies for preventing CRC–PC. In addition, early and timely immune enhancement through drugs may achieve similar effects of HFD therapy. However, it remains to be further investigated whether a fat emulsion can reduce the incidence of intraperitoneal implant metastasis in CRC patients by improving postoperative immunity through rigorous clinical trials.
